# The stability and catalytic activity of W_13_@Pt_42_ core-shell structure

**DOI:** 10.1038/srep35464

**Published:** 2016-10-19

**Authors:** Jin-Rong Huo, Xiao-Xu Wang, Lu Li, Hai-Xia Cheng, Yan-Jing Su, Ping Qian

**Affiliations:** 1 Department of Physics, University of Science and Technology Beijing, Beijing 100083, China; 2 Corrosion and Protection Center, Key Laboratory for Environmental Fracture (MOE), University of Science and Technology Beijing, Beijing 100083, China

## Abstract

This paper reports a study of the electronic properties, structural stability and catalytic activity of the W_13_@Pt_42_ core-shell structure using the First-principles calculations. The degree of corrosion of W_13_@Pt_42_ core-shell structure is simulated in acid solutions and through molecular absorption. The absorption energy of OH for this structure is lower than that for Pt_55_, which inhibits the poison effect of O containing intermediate. Furthermore we present the optimal path of oxygen reduction reaction catalyzed by W_13_@Pt_42_. Corresponding to the process of O molecular decomposition, the rate-limiting step of oxygen reduction reaction catalyzed by W_13_@Pt_42_ is 0.386 eV, which is lower than that for Pt55 of 0.5 eV. In addition by alloying with W, the core-shell structure reduces the consumption of Pt and enhances the catalytic efficiency, so W_13_@Pt_42_ has a promising perspective of industrial application.

The foreground of sustainable energy is built upon a renewable and environmentally compatible scheme of chemical-electrical energy conversion^1^,^2^. Proton exchange membrane fuel cells (PEMFCs) demonstrate much higher thermodynamic efficiency and are more environmental-friendly than conventional fossil fuel-based engines to power transportation vehicles^3^. Moreover, the high energy density, relatively low operating temperature, and minimal corrosion susceptibility make them a promising alternative for mobile and transport applications^4^. Current electrocatalysts, used as the cathodes for the oxygen reduction reaction, are typically Pt nanoparticles (NPs) on amorphous high-surface-area C^5^,^6^. The drawback of existing electrocatalyst technology is high Pt loading in fuel-cell cathodes. The limited supply and high cost of Pt remain as a grand challenge before this technology can be commercialized. Compared with bulk pure Pt catalysts, the nano-scale Pt alloys compounded with late transition metal (TM) elements in 3*d* series (TM=Co, Ni, Fe, etc.) exhibit better catalytic activity and lower cost^7^,^8^,^9^,^10^,^11^,^12^. However, the electrochemical stability of Pt–M alloy NPs is still under dispute. The tendency to dissolve in acidic solutions^13^,^14^,^15^ is attributed to the relatively low cohesive energy of Pt alloy NPs. Thus, raising the cohesive energy of Pt-based alloy NPs will improve their stability^16^,^17^. Pt–M alloy catalysts with ordered and disordered structures are both susceptible to non-noble-metal electrochemical dissolution, although the disordered phases have higher durability^18^. To seek new materials with stronger dissolution resistance in acidic solutions, core-shell bimetallic NPs have gained much attention because of their unique structure in the process of catalysis and electrocatalysis^19^,^20^,^21^. Zhang *et al*.^22^ noted that if alloy NPs were molded into core-shell structures (thin skin layers of noble metals surrounding non-noble metals), they would withstand acidic electrolytes. Moreover, the core-shell structures with Pt layer coating the non-noble metal core would exhibit a higher specific activity for oxygen reduction reaction (ORR) than pure Pt NPs^23^,^24^,^25^,^26^.

Recently, Dai *et al*.^27^ found that alloying Pt with W formed a stable Pt-enriched surface even if the concentration of W is as high as PtW_2_, because Pt has a strong surface segregation tendency in Pt-W alloys. In particular, W exhibits corrosion resistance in acidic media and has been used as an anodic material along with Pt in PEMFCs. Moreover, W can modify the electronic structure of the surface Pt and weaken the bind for oxygenated species^27^. The mass activity of PtW_2_ alloy catalysts is nearly four times higher than that of pure Pt catalysts. Also, the activity and the surface area of PtW_2_ alloy catalysts are nearly constant over 30,000 potential cycles in catalysis under the oxidizing conditions of ORR^28^, however, those of pure Pt catalysts suffer significant losses in the process. The ORR activity also highly depends on the size of the NPs. The specific activity undergoes a rapid four-fold increase as the particle size grows from 1.3 to 2.2 nm and elevates slowly with further size rises^7^,^29^,^30^,^31^,^32^. Considering the excellent catalytic activity of PtW_2_ alloy catalysts, it is necessary to study the catalytic activity of the W-Pt core-shell structure. Wang *et al*. studied the core-shell structure using semi-sphere models^33^ and sphere-like NP models^34^. The semi-sphere models reduce the computation time and characterize the structure comprehensively. The sphere-like NP model, which is solid and hollow, is adopted to increase the number of high-coordination surface sites per Pt mass and enhance catalytic activity and durability for ORR. Because we used small model size, *i.e.*, approximately 1 nm, containing 55 atoms, the computation time and cost is acceptable even though we adopt an entire ideal particle model throughout the calculations. In this article, comprehensively considering the catalytic activity and computation cost, we sampled the icosahedron W_13_@Pt_42_ core-shell structure with a diameter of approximately 1 nm as an ORR catalyst, whose surface contains twelve vertex Pt_v_ atoms and thirty edge Pt_e_ atoms. It indicates that the icosahedron W_13_@Pt_42_ core-shell structure is a promising nanocluster to replace the pure Pt NPs in ORR owing to lower Pt loading, stronger stability and higher catalytic activity.

It is important to emphasize that our work offers only a theoretical prediction of the structural effects on the catalysis properties of the W_13_@Pt_42_ core-shell. The influence of ligands is not taken into consideration, which may affect the properties in real conditions. We hope the W_13_@Pt_42_ cluster can be verified and developed by experimentalists.

## Results and Discussion

### The stability of W_13_@Pt_42_

**Figure 1:**
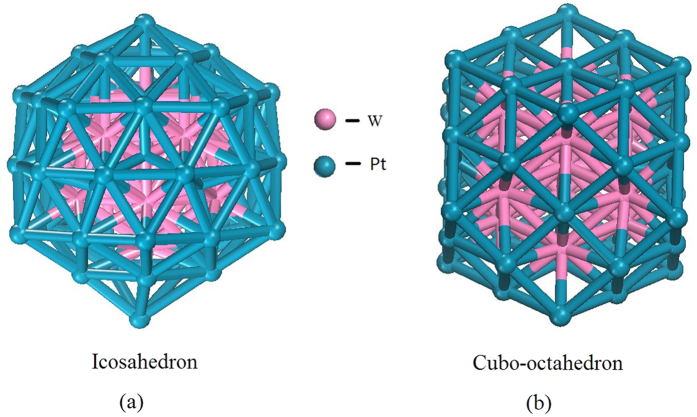
The different isomeride structures of W_13_@Pt_42_.

The cubo-octahedron and icosahedron are observed in the nanocatalysts of PEMFCs with 55 atoms^35^,^36^. Two potential structures of W_13_@Pt_42_ are shown in Fig. 1. As the result of our calculation shows, the icosahedron structure has more negative total energy than the cubo-octahedron structure (−387.24 eV *vs* −380.49 eV). It is also demonstrated that the formation of the core−shell icosahedron configuration plays a decisive role in the stability of nanoalloys with 55 atoms because of the release of strain energy, which favors the formation of nanoalloys with only one species on the surface^16^, such as Al_13_@Pt_42_[37, Co_13_@Pt_42_^38^, Ni_13_@Pt_42_^39^, Fe_13_@Pt_42_^40^ and Rh_13_@Pt_42_^41^. Thus, we here select an icosahedron core-shell W_13_@Pt_42_ cluster as the ORR catalyst, whose surface is assembled with twelve vertex Pt_v_ atoms and thirty edge Pt_e_ atoms. Furthermore, using Equation (1), we calculated the binding stability of W_13_@Pt_42_; it has a high stability in contrast to Pt_55_ (E_bind_ = −5.45 eV/atom *vs* −5.06 eV/atom). Thus, replacing the Pt_55_ cluster with the W_13_@Pt_42_ core-shell will not weaken the durability of the catalysts. To investigate the stability at room temperature, we carried out the molecular dynamics simulations at 300 K; the results indicate that the thermal stability of the structure is acceptable (the stable structures at T = 0 K and T = 300 K are shown in Figure S1).

The environmental conditions around the NPs, such as in contact with acidic solutions or adsorbing chemical species, will affect the stability and operation of the core-shell catalyst. We will investigate these effects in the following sections.

### The dissolution resistance in acidic medium

To confirm the estimation of the stability of W_13_@Pt_42_, using Equations (2) and (4), we calculate the core-shell interaction energies E_cs_ and the Pt_42_ shell dissolution potentials U_diss_ (TM_13_@Pt_42_), as presented in Table 1. The results indicate that U_diss_ and E_cs_ are enhanced compared with TM_13_@Pt_42_ (TM = Ni, Co, Fe, Al) and Pt_55_, which have been well studied^37^,^38^,^39^,^40^,^41^. The corresponding order is W_13_@Pt_42_>Al_13_@Pt_42_>Fe_13_@Pt_42_>Co_13_@Pt_42_>Ni_13_@Pt_42_>Pt_55_. Specifically, the Pt-skin layer that dissolves into the acidic solution is much weaker because there is a stronger binging and charge transfer between a W_13_ core and Pt_42_ shell. We conclude that the electrochemical stability of W_13_@Pt_42_ is favorable to act as an ORR catalyst.

**Figure 2:**
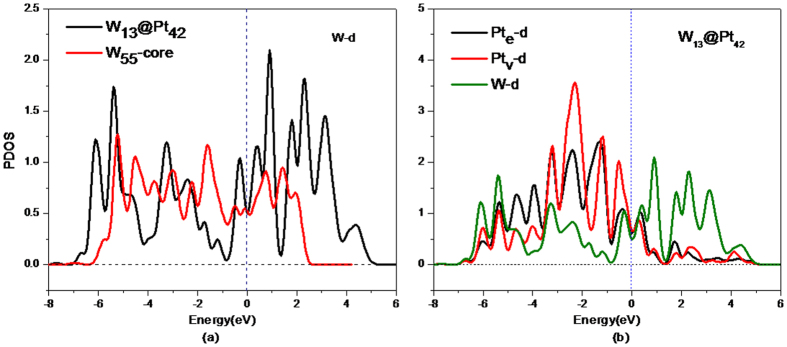
The *d*-state PDOS of the W and Pt atoms. (**a**) W atoms in W_13_@Pt_42_ and W_55_; (**b**) W and Pt atoms in W_13_@Pt_42_.

To identify the source of the stability of the core-shell W_13_@Pt_42_ in an acid solution, the partial density of states (PDOS) of the W and Pt atoms in W_55_ or W_13_@Pt_42_ are shown in Fig. 2. From Fig. 2(a), it can be observed that the W-*d* electrons in the core-shell W_13_@Pt_42_ structure distribute more discretely and occupy a larger energy scope compared with those in the W_55_ structure. As Fig. 2(b) reveals, the electron distributions of W-*d* and Pt-*d*, regarding both Pt_e_ and Pt_v_, are similar and exhibit a strong orbital hybridization. It is clear that a strong interaction exists between W-*d* and Pt_e_-*d* at 1.5 eV, 0.4 eV, −0.3 eV, −2.5 eV, −3.2 eV, −5.3 eV and −6.0 eV. Considering the W-*d* and Pt_v_-*d* states, the prominent overlaps of states emerge at −6.0 eV, −5.3 eV, −3.2 eV, −2.5 eV, −0.3 eV, 0.4 eV, 1.5 eV and 4 eV. Both imply that a tight W-Pt bond has formed. Compared with the hybridization between W-*d* and Pt-*d*, the *s*-*d* interaction between W and Pt atoms is weak and can be ignored. Therefore, the excellent stability of W_13_@Pt_42_, especially in an acidic medium, is largely attributed to the hybridization between the Pt-*d* band and the W-*d* band.

**Figure 3:**
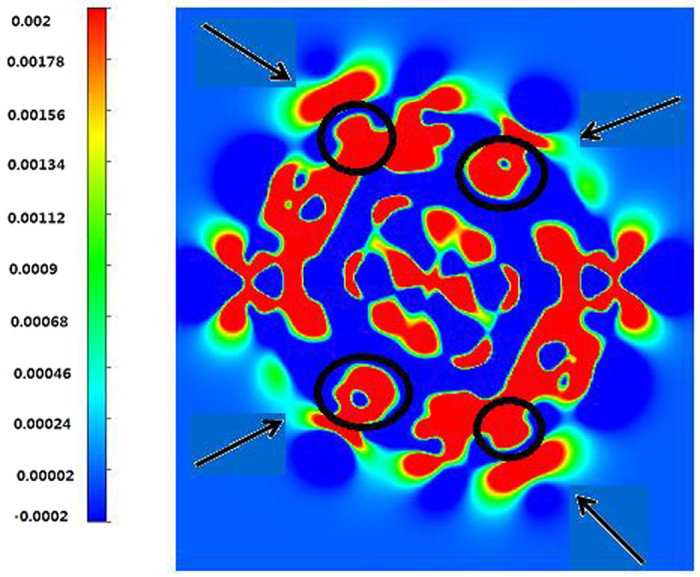
Plot of the 2D electron density difference for W_13_@Pt_42_.

To clarify the relationship between the structure stability and the charge transfer between the W core and Pt shell of W_13_@Pt_42_, the electron density difference is shown in Fig. 3. A sharp increase of the electron density mainly appears at the juncture of Pt and W atoms. It reveals an abundant charge transfer from W to Pt and verifies the existence of strong Pt-W bonds. As we have observed, the distribution of electron density difference is compatible with that of PDOS in Fig. 2.

### Adsorbate-induced structure stability test

Existing research shows that one O atom adsorbed on the Co_13_@Pt_42_ core-shell structure cannot raise Co to the surface but two O atoms would segregate a single Co atom from the surface^17^; it is not clear whether that phenomenon also occurs in the W_13_@Pt_42_ nanocluster. Once W atoms are segregated, the structural integrity of the Pt–W nanocluster is seriously degraded because W is more easily dissolved in an acidic solution^42^ than Pt within the electrode potential window of PEMFCs.

**Figure 4:**
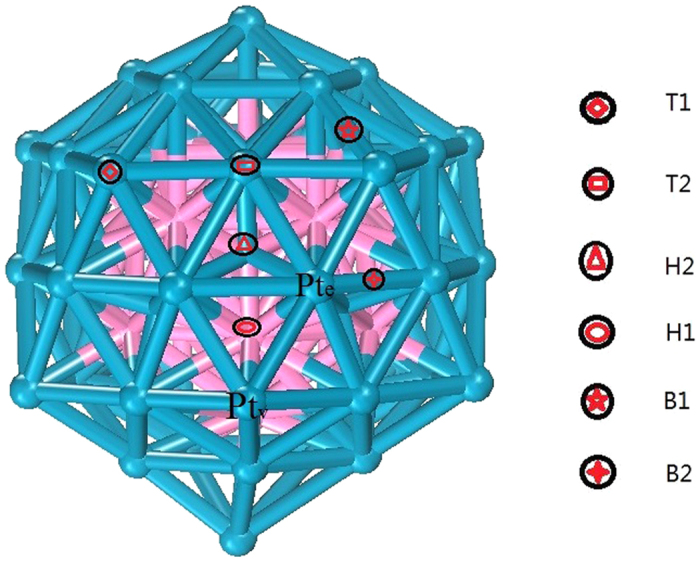
O adsorption sites of the W_13_@Pt_42_ core-shell structure.

**Figure 5:**
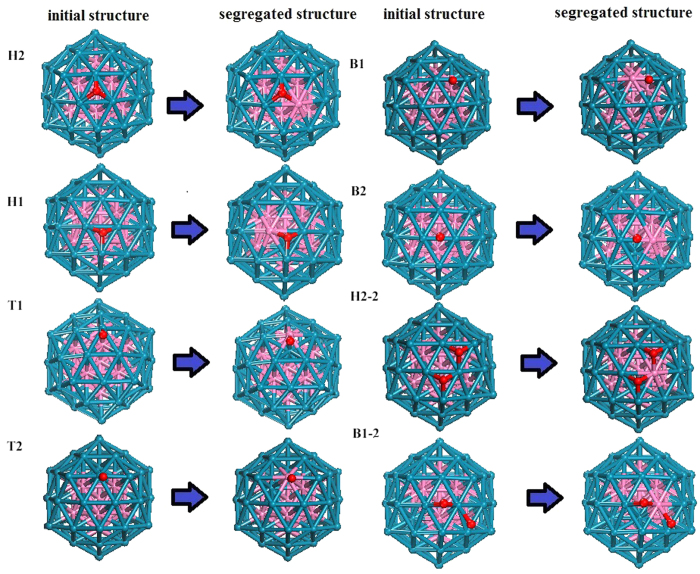
Adsorption of one or two O atoms on the W_13_@Pt_42_ core-shell structure.

For the different adsorption sites, as displayed in Fig. 4, we investigate the structure change between the initial and segregated ones in Fig. 5. Using Equation (5), we calculated the segregation energy 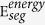 of each adsorbate cluster. After comparing with the E_seg_ of different structures, as shown in Table 2, we find that W atoms can not transfer to the surface if only one O atom is adsorbed. There is a change when the number of O atoms is two, however; 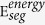 becomes a negative value, indicating that W atoms would rise to the surface. Moreover, the amount of W atoms that rise to the shell tends to increase when more O atoms are adsorbed. Because the W atoms are more easily dissolved in an acidic solution, the core-shell structure will be corroded. This result indicates that as the number of adsorbed O atoms increases, the stability of the catalyst decreases. Therefore, to prevent this phenomenon, the ORR should properly control the concentration of O atoms.

### The kinetics of ORR mechanisms

To further confirm the adsorption energy of W_13_@Pt_42_ lower than that for Pt_55_ cluster, we consider the adsorption energy of Pt_55_ for supplementary purposes. As is shown in Table S1, the adsorption energy of W_13_@Pt_42_ for both O and OH is smaller than that for Pt_55_, indicating a better catalytic activity of the core-shell W_13_@Pt_42._

### Supported and unsupported cluster structures adsorption strength

In particular, anchoring nanocatalysts on C substrates or other supports adds an additional parameter to the electrocatalyst system, as it has a more suitable adsorption energy. Taking this into consideration, the adsorption energy of supported and unsupported core-shell structures on O or OH are listed in Table S1. The supported structures on the pristine graphene or single vacancy graphene are displayed in Figure S2. As a consequence, the adsorption ability of O or OH for supported and unsupported core-shell structures is less different. The stronger interaction appears between the core-shell structure and the support instead of that between the adsorbate and the cluster.

To minimize the computational cost but maintain the scientific accuracy, we focus on the unsupported core-shell W_13_@Pt_42_. We used the fact that the structures of icosahedral Pt–Co NPs are highly symmetric, *i.e.*, all of the twenty (111) facets are symmetrically equivalent. Thus, we are able to only consider the symmetrically independent configurations of the adsorbed O atoms or OH on the surfaces.

**Figure 6:**
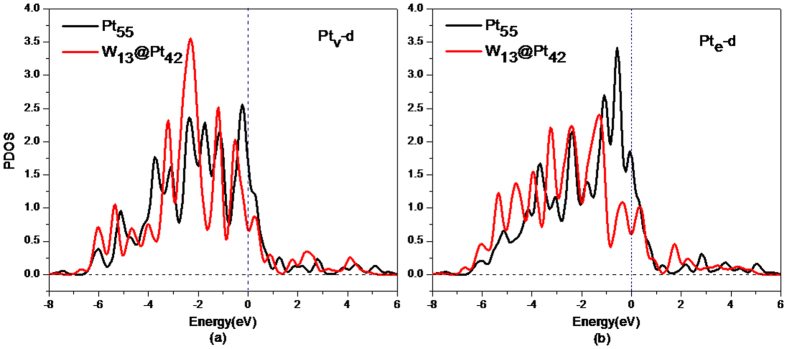
The partial electronic density of states of Pt atoms in Pt_55_ and W_13_@Pt_42_.

The adsorption energies for the Pt atoms localized in the vertex (Pt_v_) and edge (Pt_e_) sites (Fig. 4) are presented in Table 3. At different sites, such as Pt_v_ or Pt_e_, the adsorption energies of O and OH are not same. To clarify this phenomenon, the 5*d* state electronic density of states of Pt atoms in Pt_55_ and W_13_@Pt_42_ are plotted in Fig. 6. The *d*-band center of Pt atoms in W_13_@Pt_42_ shifts away from E_*F*_ compared with Pt_55_. Moreover, the *d*-band center moves towards the lower-energy range from −2.306 eV of Pt_e_ to −2.075 eV of Pt_v_; this evidence corresponds to the weaker adsorption ability for O and OH of Pt_e_. It is uncertain whether the ORR mechanism is changed from the presence of the low-coordinated atoms of nanometer size. As described by literatures^43^,^44^, we derive the effective coordination number (*N*_*eff*_) to illustrate the effect of W_13_ core. In Table 4, the *N*_*eff*_ of atoms under different chemical conditions is displayed.

The larger effective coordination number of Pt_e_ (10.5), than Pt_v_ (9.5) and Pt(111) (9) atoms corresponds to a weaker adsorption function, suggesting an increase in coordination number with the decrease in adsorptive strength, as intuitively expected. This is consistent with the interrelation of Pt_e_ and Pt_v_ on the *d*-band center, as is depicted above. The fact that the *d*-band center is not entirely predictive of the O and OH adsorption energies suggests that a more careful analysis on the surface electronic structure is necessary to explain the binding of O or OH. Thus, an analysis of Bader charges is performed.

**Figure 7:**
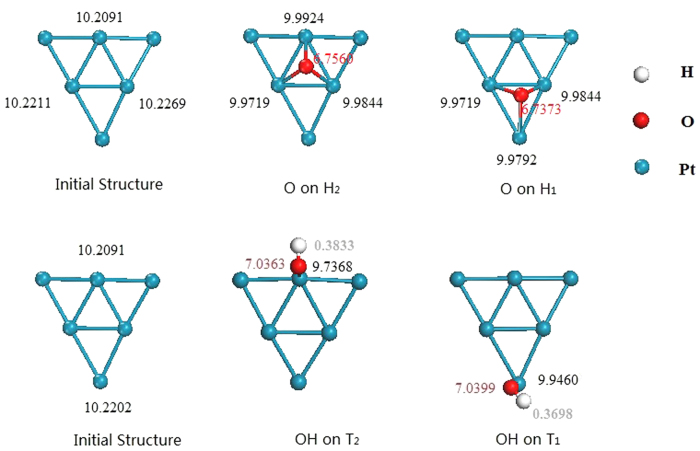
Bader charges analysis for O and OH adsorption. The unit is electrons.

Figure 7 displays the Bader charge analysis of adsorbed O and OH. When the O atoms are adsorbed on the H1 site, the electrons first transfer from W to Pt and then converge to O atoms. The calculation indicates that the three Pt atoms nearest to O display a low electropositivity. Compared with the H2 site, the electrostatic attraction between W and Pt atoms is weaker, whereas on the Pt(111) surface, the electrostatic attraction of electricity is stronger. The mechanisms of electron transfer when OH is adsorbed on Pt are similar to that of O atoms; specifically, the electron transfer is stronger on T2 than on T1. A feeble electrostatic attraction exists and develops into a Pt-W covalent bond.

### Reaction paths of ORR

Recently, a new path for ORR was proposed: OH formation in a solution comes from O and H_2_O, and the ORR on Pt(111) is essentially carried out by the O_2_ dissociation mechanism, namely, O_2_ dissociation, OH formation and H_2_O formation^3^,^45^.

**Figure 8:**
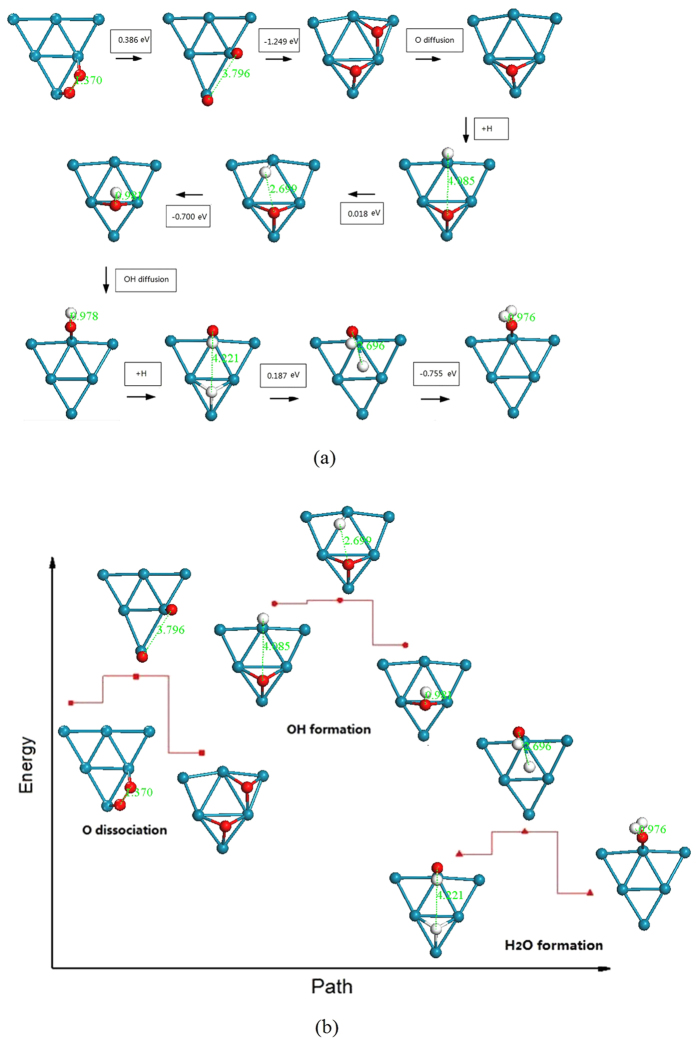
Optimized ORR path for W_13_@Pt_42_ catalysis. (**a**) Reaction barrier. The number in the box equals to the total energy difference of the neighboring two structures. The unit of the bond length is Å. (**b**) Reaction path. The Pt, O and H atoms are in blue-green, red and white, respectively.

The possible elemental reaction steps involved in the ORR which is catalyzed by a W_13_@Pt_42_ core-shell structure are shown in Figure S3; the optimal path is displayed in Fig. 8. As is shown in Fig. 8, the rate-limiting step (RDS) of the ORR mechanism is located in the O_2_ diffusion into two O atoms, with E_a_ = 0.386 eV, and is lower than that for cluster Pt55 of 0.5 eV^37^. Therefore, the path we present in this paper is more effective. It is well known that a magnitude of E_a_ < 0.75 eV is regarded as a surmountable barrier for the surface reactions at room temperatures^46^. The potential barrier forming OH from H+O is very low because the adsorption energy difference between O on the bridge and on H1 is small. However, the water and other solutions may play a considerable role in this process.

## Conclusions

In summary, the stability of W_13_@Pt_42_ core-shell structure and the ORR catalytic mechanism have been studied using first-principles calculations. Replacing the pure Pt cluster with a core-shell structure W_13_@Pt_42_ as a cathode catalyst not only lowers the cost but also provides superior stability and catalytic performance. The dissolution resistance and core-shell interaction energies in an acidic medium are the primary parameters for evaluating the durability of a catalyst. Compared with other TM_13_@Pt_42_ (TM=Ni, Co, Fe, Al), E_cs_ and U_diss_ of W_13_@Pt_42_ are more negative, which indicates better stability. We have measured the structure stability under O atom adsorption; the evidence suggests that the structure could remain stable if the O atoms concentration is limited and suitable. Moreover, we plot the electron density difference and PDOS of the structure. The electron density difference reveals that the good stability of the W_13_@Pt_42_ structure is attributed to the abundant charge transfer from core W_13_ to shell Pt. Namely, the W_13_@Pt_42_ core-shell structure is a good candidate for the ORR catalyst.

Furthermore, the reaction process and reaction barrier of ORR catalyzed by W_13_@Pt_42_ have been presented. Better catalytic activity than for nanoclusters is due to the optimal OH formation energy. The weaker adsorption energy of OH prevents the poisoning of the O-containing intermediate. These conditions favor ORR activation at room temperature.

### Computational details and method

Our calculations were performed within the density functional theory (DFT)^47^,^48^ framework, in which the generalized gradient approximation (GGA)^49^,^50^ to the exchange-correlation energy functional, as formulated by Perdew, Burke, and Ernzerhof (PBE)^49^, and the interaction potentials of the core electrons are replaced by the projector augmented wave (PAW)^51^ pseudopotential, as implemented in the Vienna ab initio Simulation Package (VASP) code^52^,^53^. We adopted the PAW method with 6*s*5*d* and s*1*d*9* valence electrons for W and Pt atoms, respectively. Kohn–Sham orbitals were expanded by plane waves up to a cut-off energy of 400 eV; ionic and electronic relaxation converged within an error of 1 × 10^−3^eV/atom, and the convergence precision was set to a force of less than 5 × 10^−2^ eV/Å. Only the gamma point was used to sample the Brillouin zone of each W_13_@Pt_42_ NP and the core-shell structure adsorbed molecule. A smearing of 0.2 eV to the orbital occupation was applied to achieve accurate electronic convergence. All atoms in our model systems were fully relaxed to obtain optimized structures.

The integration of the Brillouin-zone was performed using a 2 × 2 × 1 Monkhorst–Pack^54^,^55^ grid with Γ points for the supported metal cluster. For free metal clusters, a rectangular supercell with a size of 30 × 30 × 30 Å^3^ was employed in the calculations. For the W_13_@Pt_42_ cluster supported on graphene, an orthorhombic supercell of 14.76  × 14.76 × 31.51 Å^3^ with periodic boundary conditions was used. The choice of unit cell keeps the W_13_@Pt_42_–graphene system adsorbates approximately 10 Å apart laterally.

To analyze the structural stability of alloy clusters, the average binding energy (E_bing_) of a cluster was calculated following:


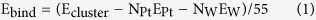


where E_cluster_, E_Pt_ and E_W_ are the total energies of Pt_55_ or W_13_@Pt_42_ clusters, Pt atoms, and Al atoms, respectively. N_Pt_ and N_W_ are the numbers of Pt and W atoms in the cluster, respectively.

The pure Pt nanostructure or Pt-base core-shell structure is more likely to dissolve when exposed to acidic media; it is significant to the durability of the catalyst. Therefore, to explain the higher stability of W_13_@Pt_42_, the core-shell interaction energy (E_cs_) and the dissolution potential of the Pt shell (U_diss_)^15^,^56^ were calculated. The interaction energy (E_cs_) was given by Equation (2):


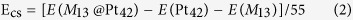


The dissolution potential of the Pt_n-m_@Pt_m_ cluster shell was calculated using Equation (3)^56^


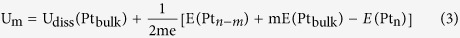


where E(Pt_bulk_) represents the total energy of bulk Pt. E(Pt_n_) and E(M_n−m_) represent the total energy of Pt_n_ or W_n−m_, respectively.

Similarly, we derived the dissolution potential of M_13_@Pt_42_ (U_diss_):


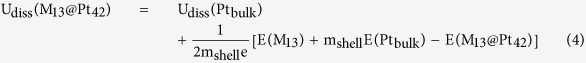


where U_diss_(Pt_bulk_) = 1.188 V^37^, m = m_shell_ = 42, and n = 55.

To determine whether the adsorption of O atoms would transfer W atoms to the surface, we calculated the segregation energy 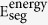 of the adsorbate cluster, which was defined as equation (5):


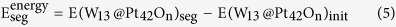


where n is the number of O atoms and 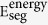
is the segregation energy. The W atoms were located in the core will rise to the shell if the segregation energy (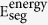) was negative. And the more negative 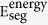 is, the more likely it will appear.

The climbing image nudged elastic band (CINEB) method^57^,^58^, a tool in the *VASP* code, is an efficient method for finding the minimum energy paths (MEPs) between a given initial and final states of a transition. For an adsorption process of a molecule, the van der Waals interaction (vdW) has an important effect. In many circumstances, the current vdW-DFT^59^,^60^ is sufficiently accurate and was used to correct the ORR energy barriers. The MEPs for ORR were obtained using NEB tools; we showed the optimized overall reaction path with the smallest potential barrier. For the rate-limiting step (RDS) of ORR, we also considered the influence of the water solvent, and the dielectric constant was set at 80^61^.

## Additional Information

**How to cite this article**: Huo, J.-R. *et al*. The stability and catalytic activity of W_13_@Pt_42_ core-shell structure. *Sci. Rep.* **6**, 35464; doi: 10.1038/srep35464 (2016).

## Supplementary Material

Supplementary Information

## Figures and Tables

**Table 1:** The calculated E_cs_ and U_diss_ (TM_13_@Pt_42_).

	Pt_55_^37^	Ni_13_@Pt_42_^39^	Co_13_@Pt_42_^40^	Fe_13_@Pt_42_^41^	Al_13_@Pt_42_^37^	W_13_@Pt_42_
E_cs_ (eV/atom)	0.816	0.933	0.995	1.087	1.101	1.233
U_diss_ (V)	−0.52	−0.53	−0.62	−0.75	−0.84	−0.953

**Table 2:** Segregation energy of a W atom under one or two adsorbed O atoms.

	Initial structure (eV)	Segregated structure (eV)	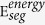 (eV)
H2	−391.754	−390.809	0.945
H1	−392.731	−392.073	0.658
T1	−391.816	−392.016	−0.200
T2	−391.554	−391.447	0.107
B1	−392.594	−392.041	0.553
B2	−392.289	−391.791	0.498
H2-2*	−397.042	−398.574	−1.532
B1-2*	−397.786	−398.354	−0.568

“*” a structure in which two O atoms are adsorbed.

**Table 3:** The adsorption energy for Pt atoms, which are local in the vertex (Pt_v_) and edge (Pt_e_) sites of Pt_55_ and W_13_@Pt_42_.

	Site	E_ads_ (O) (eV)	E_ads_ (OH) (eV)
Pt_55_	Pt_v_	−5.304	−3.376
	Pt_e_	−4.869	−4.859
W_13_@Pt_42_	Pt_v_	−4.571	−3.168
	Pt_e_	−4.309	−3.025

**Table 4:** The N_eff_ and *d*-band center *ε*_*d*_ of Pt atoms in different structures.

	W_13_@P_42_		Pt_42_		Pt_55_		Pt-(111)	
	Pt_e_	Pt_v_	Pt_e_	Pt_v_	Pt_e_	Pt_v_	Pt_e_	Pt_v_
*N*_*eff*_	10.5	9.5	10	10	7.4	7.4	9	9
*ε*_*d*_ (eV)	−2.306	−2.075	−1.949	−2.160	−1.563	−1.654	−1.947	−1.947
