# Supplementary material for: The stability and catalytic activity of W13@Pt42 core-shell structure
Source: Sci Rep. 2016 Oct 19;6:35464. doi: 10.1038/srep35464 (PMC5069721; doi:10.1038/srep35464)
Supplement: Supplementary Information [file srep35464-s1.pdf]

Supporting Information

For

The stability and catalytic activity of $W_{13}@Pt_{42}$ core-shell structure

Jin-Rong Huo[a], Xiao-Xu Wang[a], Lu Li[a], Hai-Xia Cheng[a], Yan-Jing Su[b]*, Ping Qian[a]*

[a]Department of Physics, University of Science and Technology Beijing, Beijing 100083, China

[b]Corrosion and Protection Center, Key Laboratory for Environmental Fracture (MOE)，University of Science and Technology Beijing, Beijing 100083, China

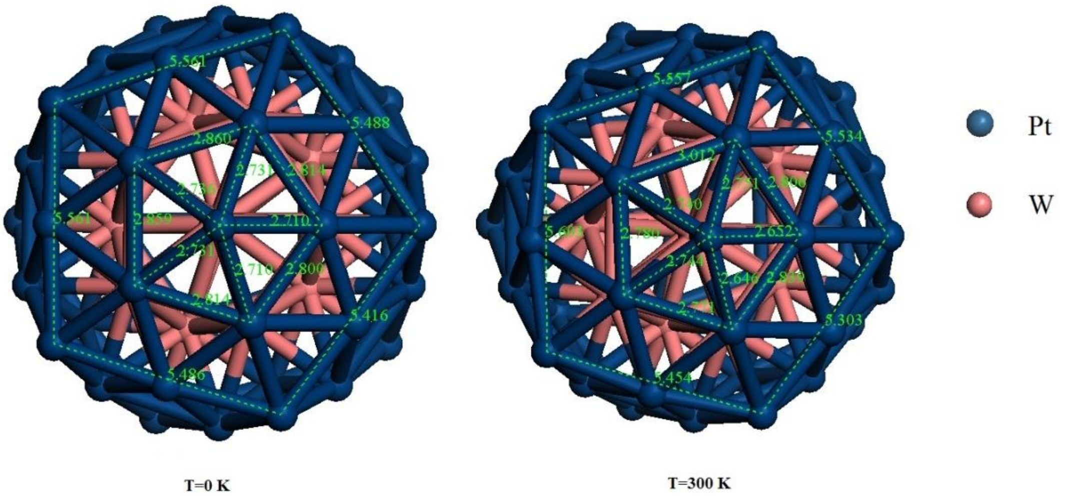

Figure S1. The stable structures of T=0 K and T=300 K. The unit of bond length is Å.

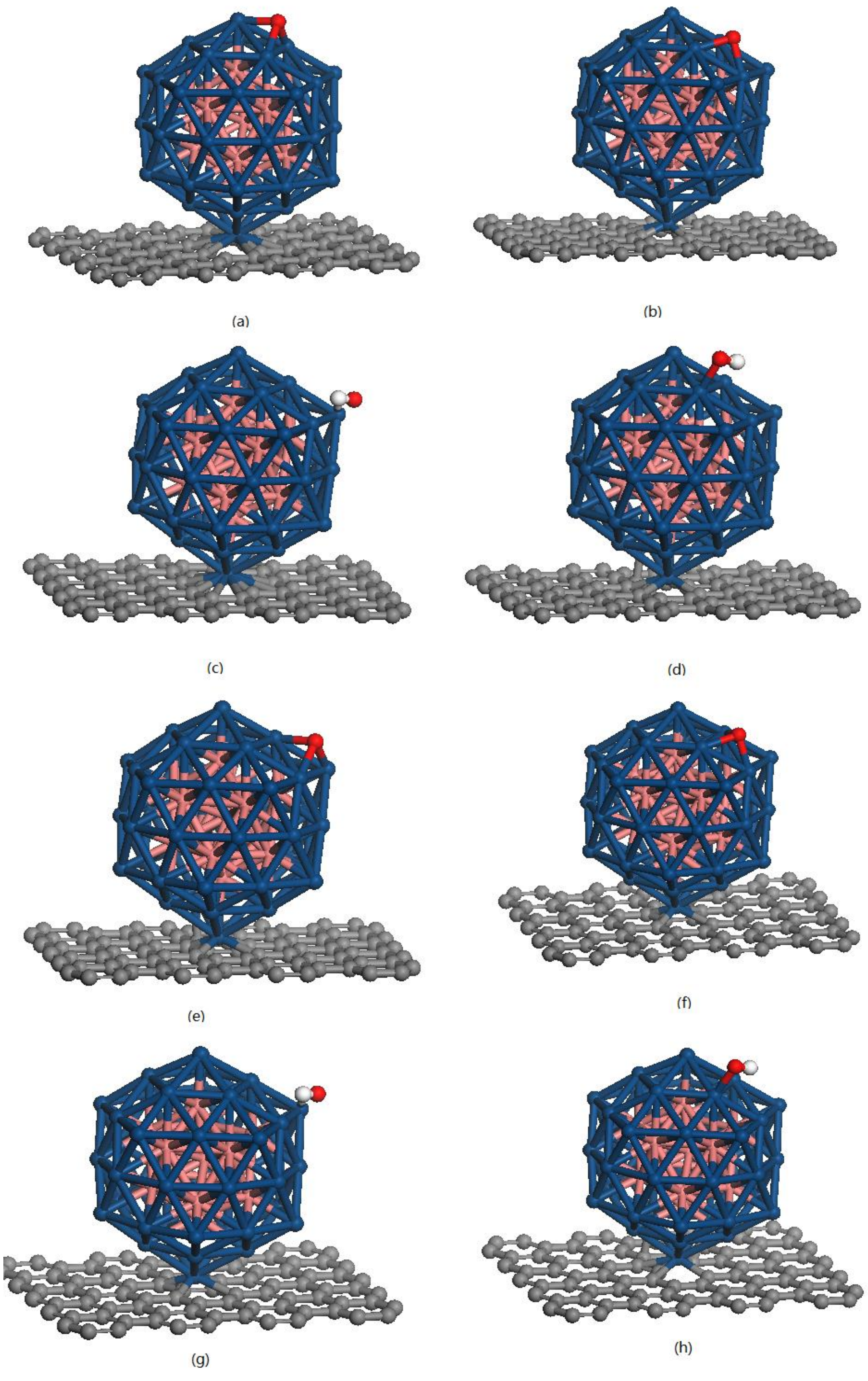

Figure S2. O atom or OH adsorbs on the supported structure of (a) G-W_{13}@Pt_{42}-O-H_1 (b) G-W_{13}@Pt_{42}-O-H_2 (c) G-W_{13}@Pt_{42}-OH-T_1 (d)

G-W_{13}@Pt_{42}-OH-T_2 (e) G-1- W_{13}@Pt_{42}-O-H_1 (f) G-1-W_{13}@Pt_{42}-O-H_2 (g) G-1-W_{13}@Pt_{42}-OH-T_1 (h) G-1- W_{13}@Pt_{42}-OH-T_2.

Table S1. The calculated adsorption energy values of E_{ads}(O) and E_{ads}(OH) on different structure.

	E_{ads}(O)(eV)		E_{ads}(OH)(eV)	
	H_1	H_2	T_1	T_2
Pt_{55}	-6.031	-6.033	-3.376	-4.859
W_{13}@Pt_{42}	-5.474	-5.585	-3.168	-3.025
G-W_{13}@Pt_{42}*	-5.551	-5.515	-3.179	-2.896
G-1- W_{13}@Pt_{42}**	-5.511	-5.441	-3.213	-2.864

*denote the structure supported by nondefective graphene.

** denote the structure supported by single vacancy graphene.

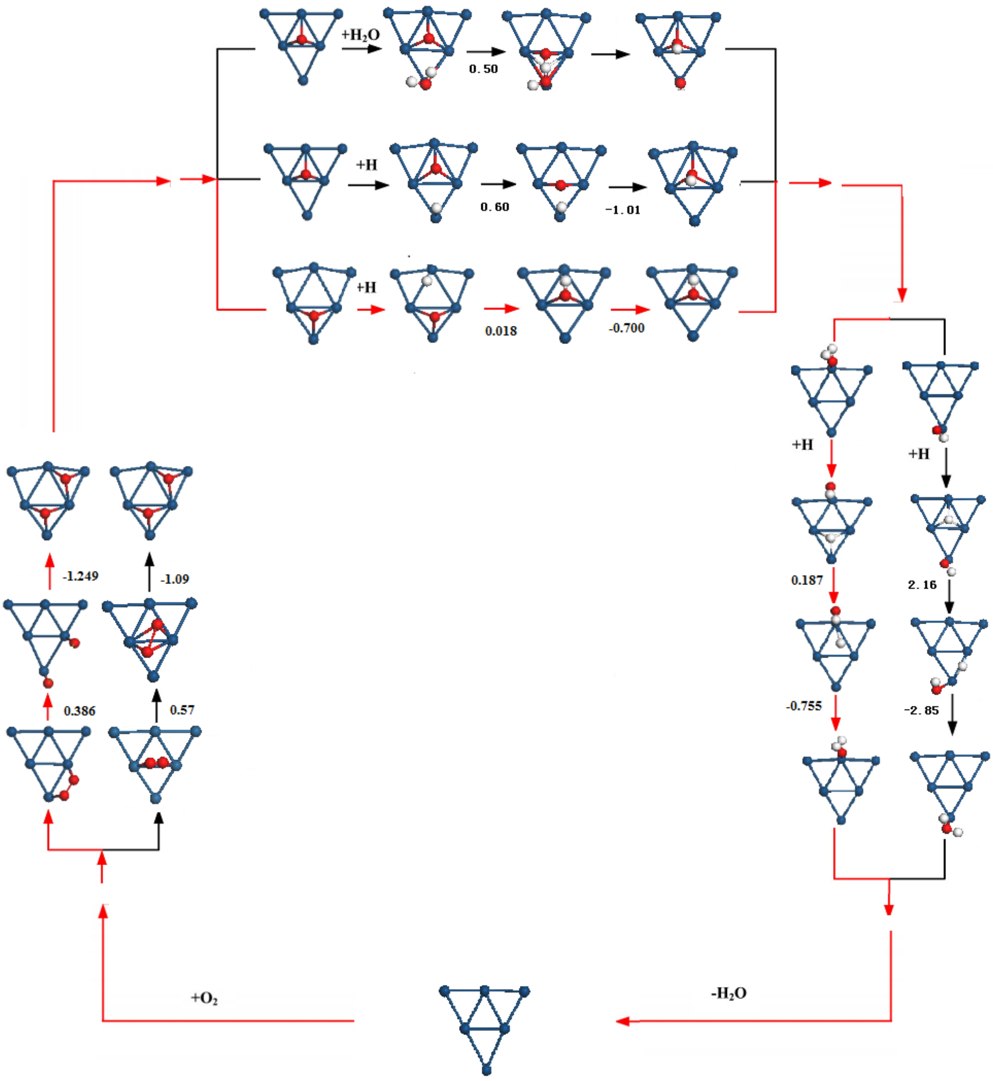

Figure S3. Reaction energy path for elemental steps of ORR on core-shell $W_{13}@Pt_{42}$. The unit of energy barrier is eV.
